# Higher rates of HBsAg clearance with tenofovir-containing therapy in HBV/HIV co-infection

**DOI:** 10.1371/journal.pone.0215464

**Published:** 2019-04-18

**Authors:** Pierre Gantner, Laurent Cotte, Clotilde Allavena, Firouzé Bani-Sadr, Thomas Huleux, Claudine Duvivier, Marc-Antoine Valantin, Christine Jacomet, Véronique Joly, Antoine Chéret, Pascal Pugliese, Pierre Delobel, André Cabié, David Rey, for the Dat’AIDS Study Group

**Affiliations:** 1 Virology Laboratory, Hôpitaux Universitaires de Strasbourg, Strasbourg University, INSERM, UMR-S U1109, Strasbourg, France; 2 Infectious Diseases Department, Croix-Rousse Hospital, Hospices Civils de Lyon, Lyon, France; 3 INSERM U1052, Lyon, France; 4 Infectious Diseases Department, CHU de Nantes, Nantes, France; 5 Infectious Diseases Department, CHU de Reims, Reims, France; 6 University Department of Infectious Diseases, Tourcoing Hospital, Tourcoing, France; 7 AP-HP-Necker Hospital, Infectious Diseases Department, Necker-Pasteur Infectiology Center; 8 Medical Center of Pasteur Institut, Necker-Pasteur Infectiology Center; Paris Descartes University, Sorbonne Paris Cité, IHU Imagine, Paris, France; 9 Infectious Diseases Department, Assistance Publique—Hôpitaux de Paris, Pitié-Salpêtrière Hospital; Sorbonne Universités, UPMC Université Paris 06, INSERM, Institut Pierre Louis d'épidémiologie et de Santé Publique (IPLESP UMRS 1136), Paris, France; 10 Infectious Diseases Department, CHU Clermont-Ferrand, Clermont-Ferrand, France; 11 Infectious Diseases Department, APHP Hôpital Bichat, Paris, France; 12 Internal Medicine Department, CHU Bicètre, France; Université Paris Descartes, Sorbonne Paris Cité, Paris, France; 13 Infectious Diseases Department, Centre Hospitalier Universitaire de Nice, Hôpital l'Archet, Nice, France; 14 Infectious Diseases Department, CHU Toulouse-Purpan, Toulouse, France; 15 Infectious Diseases Department, CHU de Martinique, Fort-de-France, France; 16 Université des Antilles EA4537 and INSERM CIC1424, Fort-de-France, France; 17 Le Trait d’Union, HIV-Infection Care Center, Hôpitaux Universitaires de Strasbourg, Strasbourg, France; Kaohsiung Medical University, TAIWAN

## Abstract

**Introduction:**

Achieving functional cure of chronic HBV infection (Hepatitis B surface antigen [HBsAg] clearance, eventually followed by acquisition of anti-hepatitis B surface antigen [Anti-HBs]) in individuals with HIV and HBV infections is a rare event. In this setting, factors related to HBV cure have not yet been fully characterized.

**Methods:**

HIV-infected individuals with chronic HBV infection enrolled in the French Dat’AIDS cohort (NCT02898987), who started combined antiretroviral (cART)-anti-HBV treatment were retrospectively analyzed for HBsAg loss and Anti-HBs seroconversion.

**Results:**

Overall, 1419 naïve-subjects received three different cART-anti-HBV treatment schedule: (1) 3TC or FTC only (n = 150), (2) TDF with or without 3TC or FTC (n = 489) and (3) 3TC or FTC as first line followed by adding/switching to TDF as second line (n = 780). Individuals were followed-up for a median of 89 months (IQR, 56–118). HBV-DNA was < 15 IU/mL in 91% of individuals at the end of the follow-up. Overall, 97 individuals cleared HBsAg (0.7/100 patient-years), of whom, 67 seroconverted for Anti-HBs (0.5/100 patient-years). A high CD4 nadir, a short delay between HBV diagnosis and treatment, a longer time on HBV therapy, an African origin and TDF-based therapy were independent predictors of HBsAg clearance (Probability of odds ratio [OR]>1, >95%) suggested by Bayesian analysis. Also, TDF-based regimen as first line (OR, 3.03) or second line (OR, 2.95) increased rates of HBsAg clearance compared to 3TC or FTC alone, with a 99% probability.

**Conclusions:**

HBsAg clearance rate was low in HIV-HBV co-infected cART-anti-HBV treated individuals, but was slightly improved on TDF-based regimen.

## Introduction

HIV-associated comorbidities remain one major public health challenge worldwide, despite the success of combined antiretroviral therapy (ART) in controlling HIV viral load and in increasing life expectancy of people living with HIV. For instance, individuals with both chronic HBV and HIV infections have an increased risk of developing both cirrhosis and hepatocellular carcinoma, leading to heightened risk of mortality [1–2]. HIV-infected individuals are at higher risk of both acquiring HBV infection (due to the shared risk of transmission factors between the two viruses) and developing chronic hepatitis B (HBV) after acute infection [3]. In European countries, chronic hepatitis B marked by a positive Hepatitis B surface antigen (HBsAg) for at least 6 months is more prevalent in HIV-infected individuals (1 to 15%) [4–7] compared to uninfected individuals (< 1%) [8]. Based on HBV infection repartition worldwide, HBV prevalence ranges from 8.5 to 28% in HIV-infected individuals in Asia or Africa [4,9–10].

Long-term virological suppression of HBV replication represents the main endpoint of current HBV therapy, while HBsAg clearance is considered an optimal endpoint [11–12]. Functional cure of chronic HBV infection, defined as HBsAg loss, followed by the acquisition of anti-hepatitis B surface antibody (Anti-HBs), is a rare event: various studies have reported HBsAg seroclearance rates ranging from 1.7 to 2.6/100 patient-years in HBV/HIV co-infected subjects undergoing different therapeutic strategies [13–15]. However, the number of participants, a short follow-up duration and the lack of real-life settings limit these data. Of note, even in individuals harboring HBV resolved serological profile, HBV viral reactivation can occur in some clinical conditions, such as immunosuppression [16] due to episomal HBV-DNA persistence in hepatocytes [17].

Nucleoside analog drugs such as lamivudine, 3TC; emtricitabine, FTC; tenofovir disoproxil fumarate, TDF; and tenofovir alafenamide, TAF, suppress HBV replication and can control HIV replication during HBV/HIV co-infection. The use of TDF-based combined antiretroviral therapy (cART) to treat this co-infection has increased over the years [18], and is now the standard for nucleoside analog backbone. However, functional cure achievement rates and related factors to HBV resolution in this setting have not yet been fully characterized [4]. In addition, quantitative HBsAg evolution has not been studied in co-infected individuals. In this study, we aimed at investigating the outcomes of a TDF and 3TC or FTC-based regimen initiation among cART-naïve chronically HBV/HIV co-infected individuals.

## Methods

### Study population and study design

**Fig 1:**
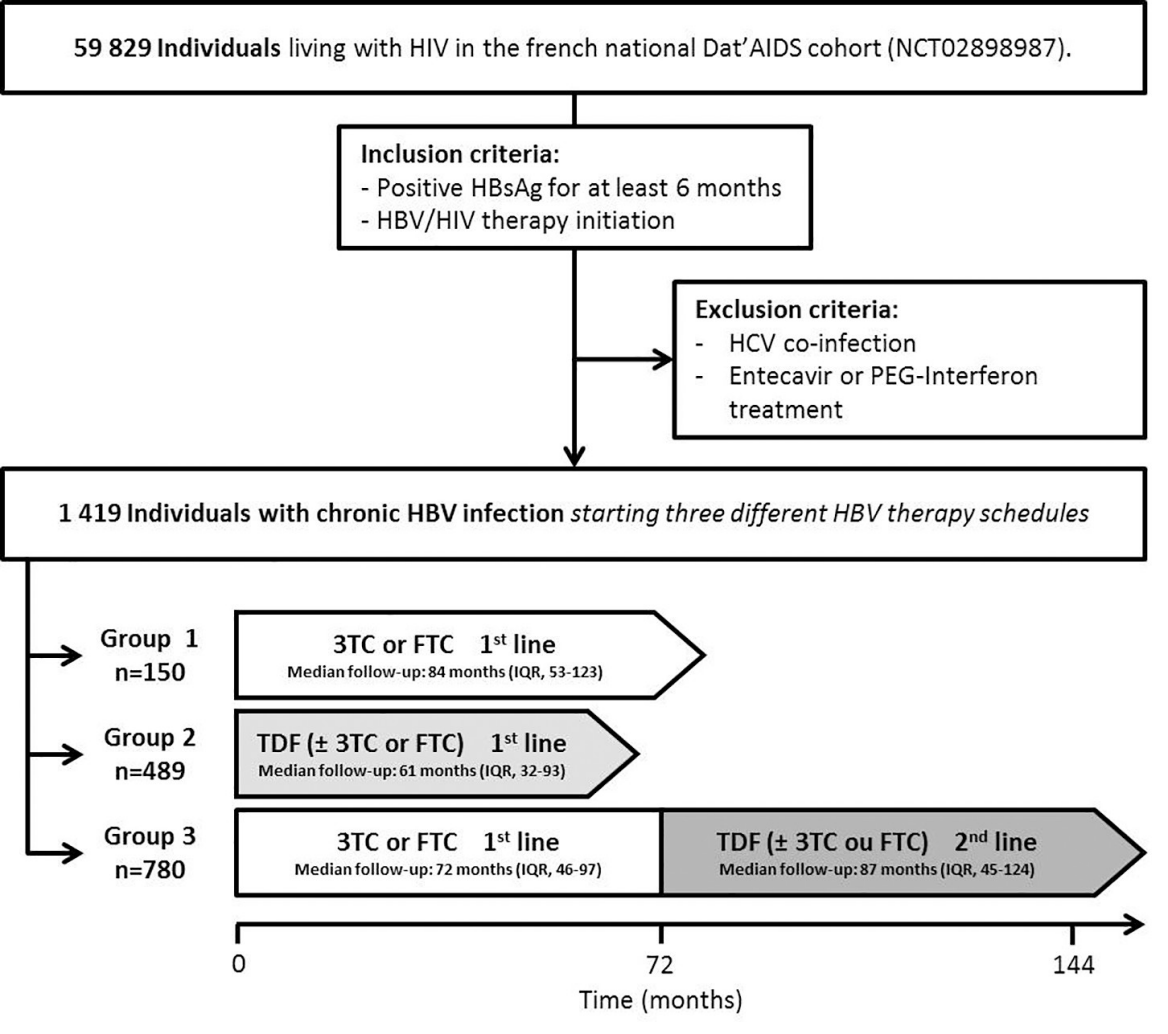
Study flowchart. Of 59829 HIV-infected individuals enrolled in the Dat’AIDS cohort between 2006 and 2016, 1419 harbored HBsAg for at least 6 months and subsequently initiated cART-anti-HBV treatment. Individuals received one of the following HBV therapy schedule: (1) 3TC or FTC only (n = 150), (2) TDF with or without 3TC or FTC (n = 489); and (3) 3TC or FTC as first line followed by adding/switching to TDF as second line with or without 3TC or FTC (n = 780). TDF, Tenofovir disoproxil fumarate; 3TC, lamivudine; FTC, emtricitabine.

cART-naïve HIV-infected individuals with chronic HBV infection, enrolled in the French Dat’AIDS cohort, who started undergoing anti-HBV-cART treatment were retrospectively analyzed (NCT02898987) [19]. This study was performed in accordance with the principles of the Declaration of Helsinki and current French legislation relating to biomedical research. As this study relied on already existing clinical data only and as there was no intervention on study participants, there was no need of an ethics committee advice according to French laws available when the study was performed. The DatAIDS cohort is registered on Clinicaltrials.gov under the identifier NCT02898987 and all included individuals provided written informed consent. Among HIV-infected individuals enrolled in the Dat’AIDS cohort, those harboring positive HBsAg for at least 6 months and then initiating HBV/HIV therapy between 2006 and 2016 were included. HCV co-infected individuals and those receiving Entecavir or PEGylated-Interferon were excluded, due to their potential interaction on HBV disease outcomes (Fig 1). Longitudinal data and variables that could influence HBV infection outcomes were recorded (HBV-DNA, HBV serology, laboratory parameters, demographic parameters, HIV-related conditions and HDV co-infection). The main study endpoints were confirmed HBsAg loss or anti-HBs seroconversion. Follow-up was censored if subjects were lost to follow-up or if they stopped anti-HBV treatment. Hepatitis B e antigen (HBeAg) was not taken into account in the analysis [20–21]. Individuals were divided into three groups based on the three HBV therapy-cART schedules: (1) those receiving 3TC or FTC only, (2) those receiving TDF with or without 3TC or FTC; and (3) those receiving 3TC or FTC as first line regimen and then switched to TDF with or without 3TC or FTC. Primary outcome measures data (HBsAg and Anti-HBs) were collected from longitudinal medical records with a frequency based on each physician’s practice.

Individuals with available quantitative HBV-DNA and quantitative HBsAg data at baseline and after more than two years of follow-up were also included in a sub study to assess the dynamics of these two parameters on HBV/HIV therapy.

Primary outcome measures were incidence rates of HBsAg loss and Anti-HBs seroconversion on two consecutive measurements. As secondary outcome measures, predictors of HBsAg loss were also assessed.

### Statistical methods

Our data were presented in two different manners. First, we **described baseline characteristics** of individuals according to the three encountered treatment schedules: (1) 3TC/FTC, (2) TDF, and (3) 3TC/FTC and then switch to TDF. Second, we **performed the statistical analysis** dividing individuals according to treatment line: (1) 3TC/FTC 1^st^ line, (2) TDF 1^st^, and (3) TDF 2^nd^ line.

HBsAg loss and anti-HBs seroconversion incidences were expressed for 100 patient-years. Kaplan-Meier curves were used to depict HBsAg loss and anti-HBs seroconversion on follow-up, according to HBV treatment regimen line (and not for the whole therapeutic schedule), with the following cases: first line 3TC or FTC; first line TDF with or without 3TC or FTC; and second line TDF with or without 3TC or FTC (the baseline for the last group corresponds to the time of switching to TDF). Survival analysis results were expressed by cumulative percentages with 95% confidence interval (CoI).

Bayesian methods were used to assess predictors of HBsAg clearance in a multivariable analysis by estimating HBsAg loss *posterior* probability (mixed logistic model) with non-informative *priors* (centered at the value of no effect, with very tight precision of 0.001) [22] and using a time-varying approach. Modeling results were expressed as Odd-ratios (OR) with credibility intervals at 95% (CrI) of HBsAg clearance. Bayesian analysis allowed calculation of the probability (Pr) of observing an OR > 1, which was found to be clinically relevant if above 95%. A predictor of HBsAg loss is thus associated with a Pr > 95%. All Bayesian analyses were performed with JAGS using the rjags package in R version 3.1.1 (R Foundation for Statistical Computing, Vienna, Austria). For individuals who received TDF as second line regimen, both time on global anti-HBV and specifically on TDF were included in the model. Of note, the whole follow-up of individuals was taken into account in the multivariable analysis.

## Results

Of 59829 HIV-infected individuals enrolled in the Dat’AIDS cohort between 2006 and 2016, 1419 (2.4%) harbored HBsAg for at least 6 months, were cART-naive and subsequently initiated cART-anti-HBV treatment. Individuals received HBV therapy according to one of the following schemes: (1) 3TC or FTC only (n = 150), (2) TDF with or without 3TC or FTC (n = 489); and (3) 3TC or FTC as first line followed by adding/switching to TDF as second line with or without 3TC or FTC (n = 780). When analyzed by treatment regimen line: 930 individuals received 3TC or FTC as first line (individuals who only received 3TC or FTC only plus those who were on 3TC or FTC as first line before switching to TDF as second line), 489 individuals received TDF as first line and 780 received TDF as second line. Subjects were primarily male (76%), had a median age of 36 years at baseline, 90% acquired both HIV and HBV by sexual transmission, and 6% of them were co-infected with hepatitis D virus (Table 1). Median follow-up was of 89 months (IQR, 56–118) but varied among groups of individuals (Fig 1). HIV virological control was achieved in 96% of individuals at the end of follow-up.

**Table 1:** Baseline characteristics of individuals with chronic HBV/HIV co-infection enrolled in the Dat’AIDS cohort.

HBV therapy schedule	Schedule 1: 3TC or FTC alone	Schedule 2: TDF ± 3TC or FTC	Schedule 3: 3TC or FTC alone then TDF ± 3TC or FTC	Total	p (Kruskal-Wallis)
n	150	489	780	1419	
HIV-1	149 (99%)	479 (98%)	776 (99%)	1404 (99%)	0.07
HIV/HBV Co-infection duration (months)	10 (6–14)	6 (6–11)	9 (6–13)	10 (6–13)	0.8
CDC stage C	49 (33%)	138 (28%)	296 (38%)	483 (34%)	0.002
CD4 nadir (/mm3)	164 (26–317)	148 (10–264)	111 (11–238)	133 (13–251)	0.9
Male	127 (84%)	325 (66%)	625 (80%)	1077 (76%)	0.004
HIV/HBV sexual transmisison	132 (88%)	451 (92%)	699 (89%)	1282 (90%)	0.5
MSM	53 (35%)	318 (65%)	285 (37%)	656 (46%)	<0.001
Age (years)	36 (31–43)	38 (32–45)	34 (29–41)	37 (30–43)	0.9
African native	54 (36%)	116 (24%)	270 (35%)	440 (31%)	0.01
HDV	4 (3%)	37 (8%)	44 (6%)	85 (6%)	0.9
Delay between chronic HBV diagnosis and treatment (months)	7 (7–8)	6 (6–7)	7 (6–7)	7 (6–7)	0.5
HIV-RNA (log10 copies/mL)	4.7 (3.8–5.0)	4.7 (4.1–5.3)	4.8 (4.1–5.3)	4.8 (4.0–5.2)	0.9
CD4 T cells (/mm3)	301 (200–420)	250 (129–380)	253 (120–378)	257 (131–382)	0.7
HBV-DNA (log10 copies/mL)	3.6 (3.4–4.2)	3.7 (3.4–4.2)	3.8 (3.3–4.1)	3.7 (3.4–4.1)	0.5

Results are expressed as number and frequency (%) or median value with interquartile range (IQR). MSM, Men who have Sex with Men; TDF, Tenofovir disoproxil fumarate; 3TC, lamivudine; FTC, emtricitabine.

HBV infection related parameters (HBsAg, Anti-HBs and HBV-DNA) were routinely assessed at a median frequency of 24 weeks (IQR, 10–54). Overall, 97 (6.8%) individuals cleared HBsAg (0.7/100 patient-years), of whom, 67 (4.7%) seroconverted for Anti-HBs (0.5/100 patient-years). No occult HBV infection was documented in these individuals, as they all were HBV-DNA negative. HBsAg clearance occurred in 25, 19 and 53 individuals in the different therapeutic schedules 1, 2 and 3 at a median time of 73, 45 and 137 months, respectively. The corresponding median time of HBsAg clearance in group 3 as of 2^nd^ line TDF regimen was of 61 months.

**Fig 2:**
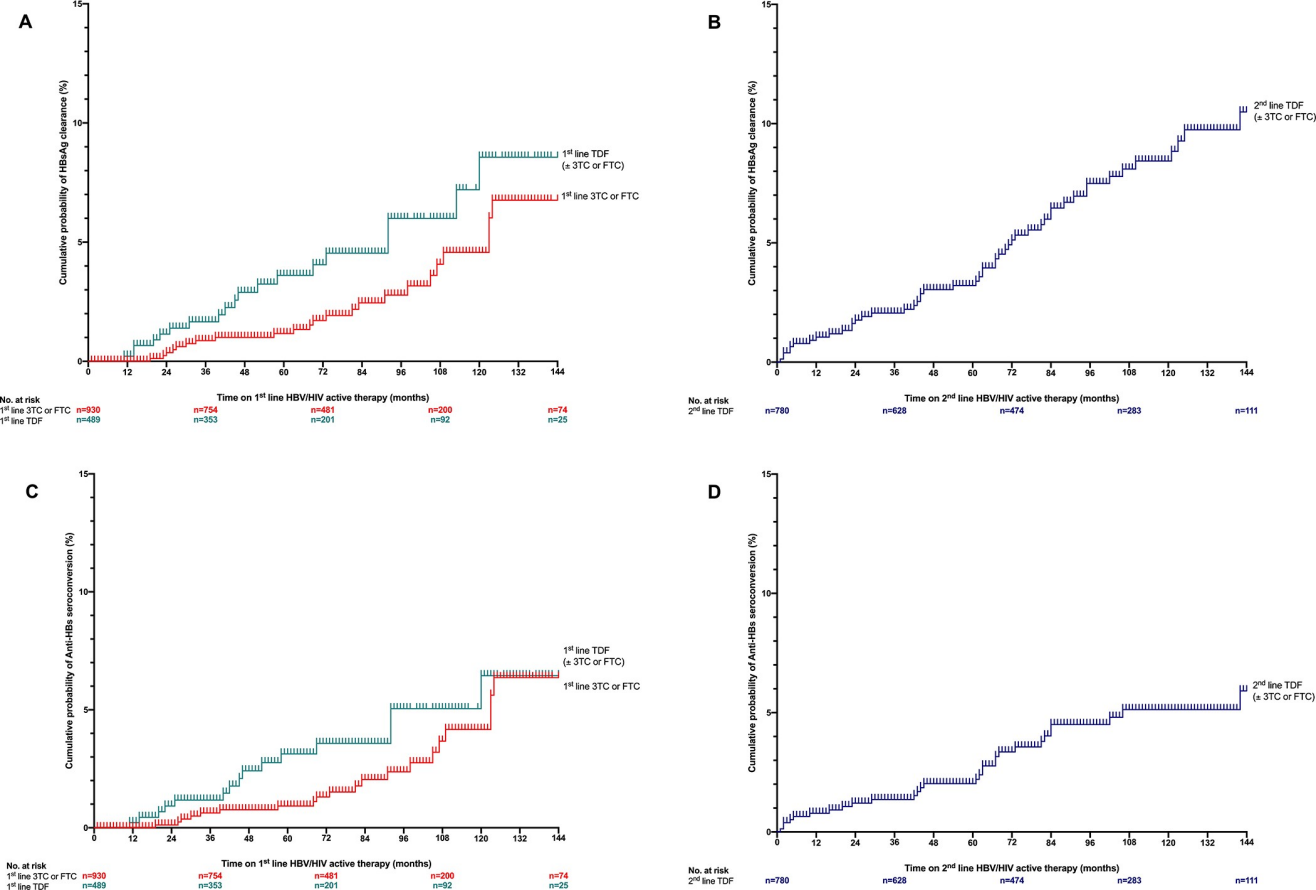
Kaplan-Meier analysis of HBsAg clearance and anti-HBs seroconversion. Kaplan-Meier analysis of HBsAg clearance on 1^st^ line regimen (A): 3TC or FTC-based regimen (n = 930, corresponding to the addition of the follow-up of individuals who only received FTC or 3TC [n = 150], plus the first part of the follow-up of those who then switched to TDF containing regimen [780]), or TDF with or without 3TC or FTC-based regimen (n = 489); and 2^nd^ line regimen (B): TDF with or without 3TC or FTC-based regimen (n = 780, corresponding to the second part of the follow-up of those who first received 3TC or FTC and then TDF). The corresponding Kaplan-Meier analyses of Anti-HBs seroconversion are depicted in (C) for 1^st^ line regimen and (D) for 2^nd^ line regimen. TDF, Tenofovir disoproxil fumarate; 3TC, lamivudine; FTC, emtricitabine.

By Kaplan-Meier analysis, HBsAg clearance rate was higher on TDF-containing regimen (*versus* 3TC or FTC alone) as 1^st^ line regimen (Fig 2A). For instance, after 72 months (half individuals of the global cohort was still on follow-up at month 72) HBsAg loss rate was; 1.7% (95% CoI, 1.2–2.2) with 1^st^ line 3TC or FTC-based regimen, 4.0% (95% CoI, 3.0–5.1) with 1^st^ line TDF with or without 3TC or FTC-based regimen. Of note, HBsAg clearance rate at month 72 was of 5.1% (95% CoI, 4.2–6.0) in individuals switching to TDF as 2^nd^ line (Fig 2B), and thus similar to 1^st^ line TDF. HBsAg clearance incidence was of 0.4/100 patient-years with 1^st^ line 3TC or FTC-based regimen, 0.8/100 patient-years with 1^st^ line TDF-based regimen and 0.9/100 patient-years with 2^nd^ line TDF-based regimen. We also observed a trend toward higher rates of anti-HBs seroconversion on TDF-containing regimen before month 96, either when used as 1^st^ (Fig 2C) or 2^nd^ line (Fig 2D).

A high CD4 nadir, a short delay between HBV diagnosis and treatment, a longer time on HBV therapy, an African origin and TDF-based therapy were independent predictors of HBsAg clearance by multivariable Bayesian analysis in our global cohort (Table 2 and S1 Table). Of note, TDF-based regimen as 1st line or 2nd line increased rates of HBsAg clearance at month 72 when compared to 3TC or FTC alone as 1st line with a > 95% probability, as suggested by Bayesian analysis. Furthermore, when repeating the multivariable analysis on a subset of 259 individuals with available quantitative HBsAg data (S1 Table), lower HBsAg levels at baseline were also associated independently with higher rates of HBsAg clearance (OR, 0.94, 95% CrI [0.89–0.99], Pr = 96%).

**Table 2:** Bayesian predictors of HBsAg clearance on HIV/HBV therapy.

Variable	OR	95% CrI	Pr [OR] < or > 1
Delay between HBV diagnosis and treatment (per 1-month increment)	0.95	0.91–1.00	97%
Time on HBV therapy (per 1-month increment)	1.08	1.04–1.13	100%
African origin (*versus* caucasian)	2.32	1.28–3.97	99%
TDF 1st line with or without 3TC or FTC (*versus* 3TC or FTC 1st line)	3.03	1.41–5.02	100%
TDF 2nd line with or without 3TC or FTC (*versus* 3TC or FTC 1st line)	2.95	1.37–5.53	96%
CD4 Nadir (per 100-/mm3 increment)	1.08	0.96–1.20	95%

TDF, Tenofovir disoproxil fumarate; 3TC, lamivudine; FTC, emtricitabine; OR, Odd-ratio; CrI, credibility interval; Pr, probability.

**Fig 3:**
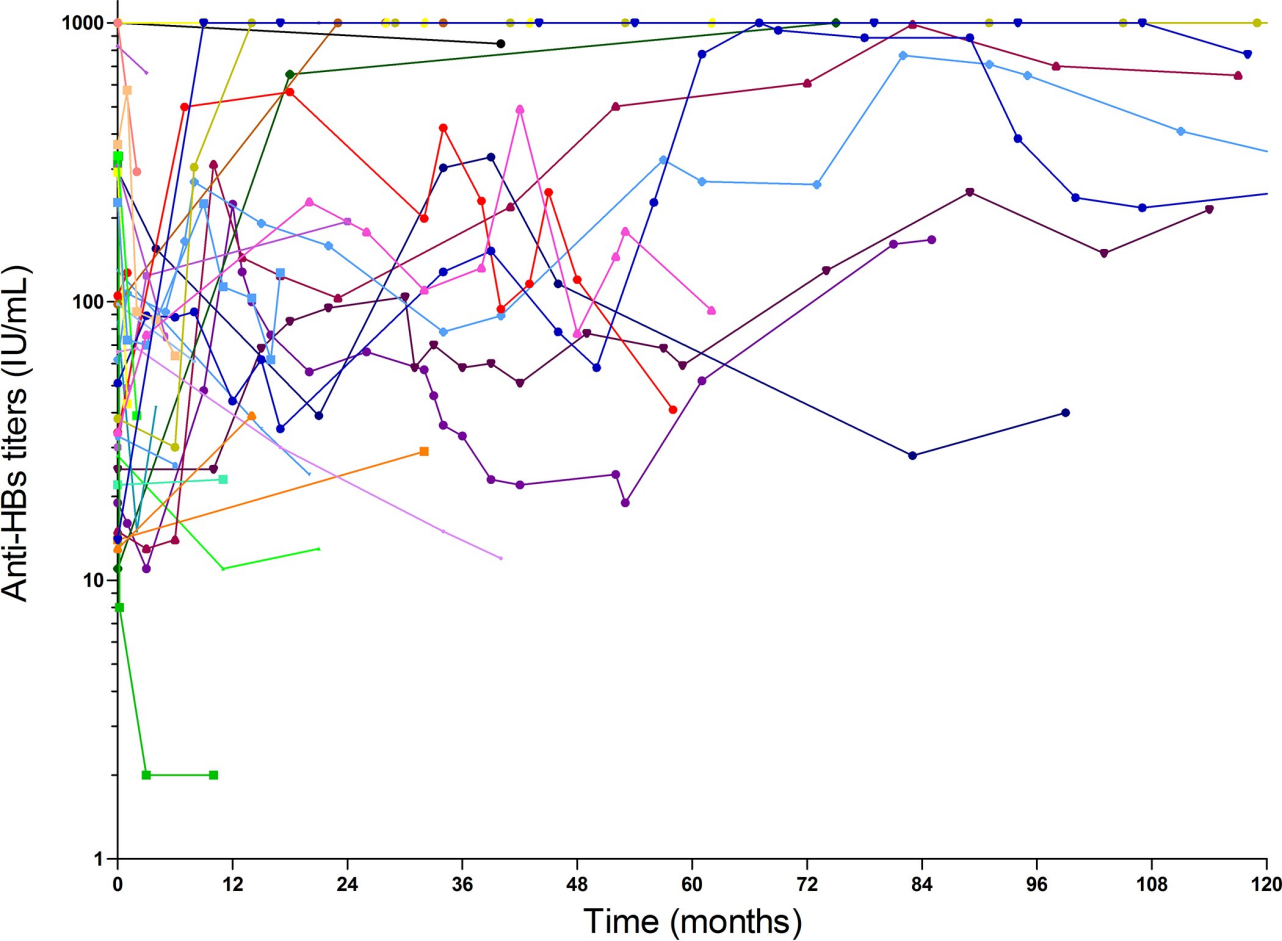
Anti-HBs titers dynamic after seroconversion. Evolution of Anti-HBs titers was depicted in the subset of 67 individuals who seroconverted for Anti-HBs on HBV therapy.

In a subset of individuals with available longitudinal follow-up of quantitative HBsAg (n = 259), HBsAg at baseline was of 2511 IU/mL (IQR, 475–5230). A slow decrease in HBsAg serum levels with a mean decrease of -1 IU/mL per year was observed on treatment. Median HBV-DNA was of 3.64 log IU/mL at baseline, and was < 15 IU/mL in 91% of individuals at the end of follow-up. Among Anti-HBs seroconverters (n = 67), median Anti-HBs titer at seroconversion was 62 IU/L (IQR, 22–296). Following seroconversion, anti-HBS titers also increased over time with a mean increase of 31 IU/L per year (Fig 3).

## Discussion

Chronic HBV co-infection burden among HIV-infected individuals results in higher rates of mortality compared to mono-infected subjects [1–2] This study in a real-life large cohort of HBV/HIV co-infected individuals showing that HBV functional cure rate remains very low, i.e., <1/100 patient-years under long term cART, including anti-HBV treatment.

Although a TDF-based regimen has not been previously associated with a faster HBsAg level decline [23], we identified this regimen as a major predictor of HBsAg clearance, with an approximately 2-fold higher rate of HBs Ag loss compared to 3TC or FTC only-based-treatments. Of note, in our study TDF was associated with 3TC or FTC in some individuals, and the duration of this association could vary among individuals.

This result is consistent with higher anti-viral efficacy in terms of achieving HBV-DNA blood suppression with TDF compared to 3TC in HBV/HIV co-infected individuals, either as first line regimen [24] or as salvage therapy [25]. Owing to the above stated effects and due to resistance issues [26], 3TC and FTC alone should not be used as part of anti-HBV viral activity, in HBV/HIV co-infected individuals. However, rates of HBsAg seroclearance in our study were lower than previously reported rates with PEGylated-Interferon (3 to 7 /100 patient-years) [11].

When compared to other cohorts of HBV/HIV co-infected individuals, HBsAg clearance and anti-HBs seroconversion rates that we observed are lower [13–15]. The large proportion of individuals taking 3TC of FTC only for a long period could partly explain this finding. However, HBsAg loss rates among TDF recipients in our cohort were also lower than the observed loss rates in HBV mono-infected (1.2/100 patient-years) [27] or HBV/HIV co-infected (3.6/100 patient-years) [28] TDF-receiving individuals.

Altogether, these results further reinforce the recommendation of using a TDF-based regimen in HBV/HIV co-infection [11]. Whether the use of the newly commercialized form of tenofovir (i.e. TAF) has a different impact on chronic HBV outcomes or not, is unknown yet and remains to be assessed in long-term follow-up studies [29–30].

Moreover, we identified other predictors of HBsAg loss, such as a prompt initiation of HBV therapy, and HBV treatment duration, in accordance with previous reports [31–32]. However, the delay between diagnosis and treatment initiation included in the model might not reflect the total duration since HBV infection, as HBV is largely undiagnosed in some key populations [33]. CD4+ T cell count has also been previously identified to negatively correlate with HBsAg clearance [34]. This result further emphasizes our finding that a high CD4+ T cell nadir correlates with higher rates of HBsAg clearance. Finally, we observed that an African origin was linked to a better prognosis of achieving HBV functional cure. This might be related to HBV genotype [35]. However, we could not assess this hypothesis as HBV genotype was poorly documented in our cohort.

We also found a very slow decline in quantitative HBsAg level on treatment. As it has previously been found to be an important predictor of HBsAg seroclearance in HIV/HBV co-infection [36–38], it could also be a prognostic factor of HBV functional cure.

Our study has several limitations inherent to its retrospective design. Indeed, some medical records can be partly incomplete, and some variables that could have an impact on HBV-active cART, such as HBV genotype, are scarce. Moreover, we could not accurately estimate the time between HIV or HBV infection and treatment initiation, as medical charts only recorded dates of each infection diagnosis. However, it represents real-life data, on a large cohort of individuals followed for more than seven years. HBeAg was not taken into account in the present analysis, as it was poorly documented in our cohort, and thus could be a limitation of our study. However, HBeAg may have a limited impact on HBsAg clearance as: (1) high rates of precore mutants are observed in France [20], and (2) HBsAg loss has been described to occur at same rates between HBeAg-positive and -negative individuals [21].

In conclusion, HBsAg clearance rates were low while on HBV therapy; higher CD4 nadir, rapid initiation of HBV therapy, mainly with TDF-based regimen, could improve HBsAg clearance and Anti-HBs seroconversion. Quantitative HBsAg decreased slowly but significantly, and therefore could be monitored as a prognosis factor of HBV clearance.

## Supporting information

S1 Table Bayesian Multivariable analysis details. Pr, Probability; OR, Odd-ratio; MSM, Men who have sex with men; FTC, emtricitabine; 3TC, lamivudine; TDF, tenofovir disoproxil fumarate.
(DOCX)
