# Supplementary material for: Higher rates of HBsAg clearance with tenofovir-containing therapy in HBV/HIV co-infection
Source: PLoS One. 2019 Apr 18;14(4):e0215464. doi: 10.1371/journal.pone.0215464 (PMC6472771; doi:10.1371/journal.pone.0215464)
Supplement: S1 Table — Pr, Probability; OR, Odd-ratio; MSM, Men who have sex with men; FTC, emtricitabine; 3TC, lamivudine; TDF, tenofovir disoproxil fumarate. (DOCX) [file pone.0215464.s001.docx]

S1 Table. Bayesian multivariable analysis details

|  | **OR** | **2.5%** | **97.5%** | **Pr(OR > or < 1)** | **Pr(OR > or < 1) > 95% ?** |
| --- | --- | --- | --- | --- | --- |
| **Model #1: Global cohort (n=1419)** | | | | | |
| Age (per 1-year increment) | 1.017992e+00 | 9.936967e-01 | 1.042196573 | 93% | No |
| Sexe |  |  |  |  |  |
| Male | 1 |  |  |  |  |
| Female | 9.929607e-01 | 4.449780e-01 | 1.899857007 | 42% | No |
| Ethnicity |  |  |  |  |  |
| Caucasian | 1 |  |  |  |  |
| African | 2.322433e+00 | 1.288749e+00 | 3.970533533 | **99%** | **Yes** |
| Asian | 9.142727e-01 | 9.712176e-02 | 2.786479548 | 34% | No |
| Other | 1.500877e+00 | 5.612509e-01 | 3.050932134 | 77% | No |
| HIV/HBV acquisition |  |  |  |  |  |
| Heterosexual | 1 |  |  |  |  |
| MSM | 1.413739e+00 | 7.505706e-01 | 2.471179942 | 83% | No |
| Intraveanous drug use | 6.449054e-01 | 1.426542e-02 | 2.744491216 | 19% | No |
| Other | 7.656048e-01 | 2.417123e-01 | 1.690884858 | 22% | No |
| HIV infection |  |  |  |  |  |
| HIV-1 | 1 |  |  |  |  |
| HIV-2 | 2.405506e+00 | 4.789453e-02 | 10.271678568 | 90% | No |
| HIV-1/HIV-2 | 1.881744e+01 | 2.411983e-01 | 100.703341509 | 62% | No |
| HIV-RNA at baseline  (per-1 log copies/mL increment) | 1.000960e+00 | 9.982553e-01 | 1.003759696 | 75% | No |
| HBV-DNA at baseline  (per 1-log IU/mL increment) | 9.889345e-01 | 7.604884e+00 | 1.230037415 | 69% | No |
| CD4 at baseline  (per 100/mm3 increment) | 1.130356e+00 | 9.971083e-01 | 1.265951387 | 94% | No |
| CD4 Nadir (per 100/mm3 increment) | 1.08744805 | 9.613166e-01 | 1.209289932 | **95%** | **Yes** |
| CDC stage |  |  |  |  |  |
| A | 1 |  |  |  |  |
| B | 1.692962e+00 | 8.809689e-01 | 2.918397798 | 94% | No |
| C | 1.223765e+00 | 6.842983e-01 | 2.033416240 | 72% | No |
| HDV co-infection |  |  |  |  |  |
| No | 1 |  |  |  |  |
| Yes | 1.340730e+00 | 4.274843e-01 | 2.923221896 | 66% | No |
| HIV/HBV co-infection duration  (per 1-month increment) | 9.572312e-01 | 9.039452e-01 | 1.009704156 | 55% | No |
| Treatment duration  (per 1-month increment) | 1.089625e+00 | 1.048524e+00 | 1.139939450 | **100%** | **Yes** |
| Delay between treatment and diagnosis of chronic HBV infection  (per 1-month increment) | 9.505257e-01 | 9.193392e-01 | 1.001848534 | **97%** | **Yes** |
| Anti-HBV drug regimen |  |  |  |  |  |
| FTC or 3TC as 1st line | 1 |  |  |  |  |
| TDF with or without FTC or FTC as 1st line | 3.030156e+00 | 1.411731e+00 | 5.023316782 | **100%** | **Yes** |
| TDF with or without FTC or FTC as 2nd line | 2.955082e+00 | 1.373630e+00 | 5.536114369 | **96%** | **Yes** |
| **Model #2: Subset of individuals with quantitative HBsAg available (n=259)** | | | | | |
| Same predictors, plus: |  |  |  |  |  |
| HBsAg at baseline  (per 10 IU/mL increment) | 9.439465e-01 | 8.900171e-01 | 9.922355e-01 | **96%** | **Yes** |
